# Assessing Concurrent Adherence to Combined Essential Medication and Clinical Outcomes in Patients With Acute Coronary Syndrome. A Population-Based, Real-World Study Using Group-Based Trajectory Models

**DOI:** 10.3389/fcvm.2022.863876

**Published:** 2022-05-25

**Authors:** Clara L. Rodríguez-Bernal, Francisco Sánchez-Saez, Daniel Bejarano-Quisoboni, Isabel Hurtado, Anibal García-Sempere, Salvador Peiró, Gabriel Sanfélix-Gimeno

**Affiliations:** ^1^Health Services Research Unit, The Foundation for the Promotion of Health and Biomedical Research of Valencia Region (FISABIO), Valencia, Spain; ^2^Red de Investigación en Servicios de Salud en Enfermedades Crónicas (REDISSEC), Valencia, Spain

**Keywords:** concurrent adherence, concomitant medications, acute coronary syndrome, group-based trajectory models, clinical outcomes, real-world data, population-based cohort

## Abstract

**Aim:**

Adherence to multiple medications recommended for secondary prevention of cardiovascular conditions represents a challenge. We aimed to identify patterns of concurrent adherence to combined therapy and assess their impact on clinical outcomes in a cohort of patients with acute coronary syndrome (ACS).

**Methods:**

Population-based retrospective cohort of all patients discharged after hospitalization for ACS (2009–2011), prescribed ≥3 therapeutic groups within the first month. We assessed monthly concurrent adherence (≥24 days of medication out of 30) to ≥3 medications during the first year, and patterns were identified through group-based trajectory models. A composite clinical outcome during the second year was constructed. The association between adherence patterns and traditional refill adherence metrics [e.g., the proportion of days covered (PDC)], and outcomes were assessed through a multivariable Cox proportional hazards model.

**Results:**

Among 15,797 patients discharged alive, 12,057 (76.32%) initiated treatment with ≥3 therapeutic groups after discharge. We identified seven adherence trajectories to ≥3 medications: Adherent (52.94% of patients); Early Gap (6.64%); Middle Gap (5.67%); Late Decline (10.93%); Occasional Users (5.45%); Early Decline (8.79%); Non-Adherent (9.58%). Compared to the Adherent group, patients belonging to Early Gap (HR:1.30, 95%CI 1.07;1.60), Late decline (hazards ratio (HR): 1.31, 95% CI 1.1; 1.56), and Non-Adherent trajectories (HR: 1.36, 95% CI 1.14; 1.63) had a greater risk of adverse clinical outcomes, which was also different to the risk ascertained through concurrent PDC < 80 (HR: 1.13, 95% CI 1.01; 1.27).

**Conclusion:**

Overall, seven adherence trajectories to ≥3 drugs were identified, with three distinct adherence patterns being at higher risk of adverse outcomes. The identification of patterns of concurrent adherence, a more comprehensive approach than traditional measurements, may be useful to target interventions to improve adherence to multiple medications.

## Graphical Abstract

Our paper at a glance: assessing concurrent adherence to combined essential medication and clinical outcomes in acute coronary syndrome patients. A population-based, real-world study using group-based trajectory models.
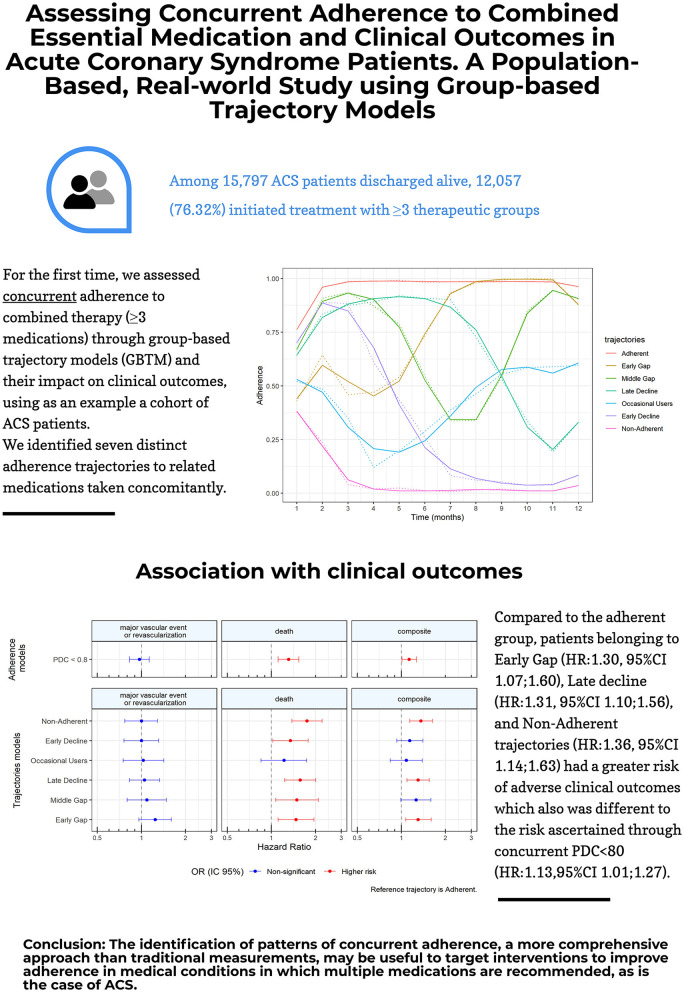

## Introduction

Physicians treating patients with cardiovascular chronic conditions (such as hypertension or coronary artery disease) often prescribe multiple medications to treat a single disease, as recommended in clinical guidelines (1–5), but adherence to this more complex therapeutic regime is often inadequately captured by assessing adherence to an individual agent or drug class (6).

In the case of patients with acute coronary syndrome (ACS), international guidelines recommend the combined use of antiplatelets, beta-blockers, angiotensin-converting enzyme (ACE) inhibitors/angiotensin II receptor blockers (ARB), and statins for the secondary prevention of this condition (3–5). Several studies have reported sub-optimal adherence to essential medications after an ACS (7–10). However, most of these studies have assessed drug classes individually. This might not reflect accurately the extent of adherence (or lack of it) to combined therapy as a whole, as regime complexity may decrease medication adherence (11–14). Therefore, it is plausible that real-world adherence to medication for any cardiovascular chronic condition requiring multiple concurrent drugs, such as ACS, is even lower than the already suboptimal figures analyzing individual drug classes (6). This may also have an impact on the accuracy of the estimates when assessing the relationship between adherence and clinical outcomes among these patients.

Furthermore, improving medication adherence is a challenge that requires methods that can reliably identify and predict when non-adherence could occur in order to customize interventions. To our knowledge, only two studies have concurrently assessed adherence to multiple therapies (statins, beta-blockers, and ACE/ARB) and tried to elucidate its relationship with clinical outcomes (all-cause mortality) (15, 16). The population groups of these studies were also conducted in patients with ACS and both used conventional measures of adherence. One study was set in the US and used the proportion of days covered (PDC) (15), while the other was a population-based, nationwide study set in Taiwan and used medication possession ratio (MPR) (16) to assess adherence.

However, grouping the patients based on a dichotomic measure may mask the changes in patients' refills, ignoring the dynamic phenomenon of adherence (17). The use of group-based trajectory models (GBTM) to measure adherence allows the identification of subgroups of patients with similar patterns of medication refill and shows the different trends in easily understandable graphics (18–21). Evidence shows that GBTM summarizes in a better way medication adherence than PDC and has better predictive accuracy on clinical outcomes (22, 23).

Therefore, adherence to therapy when a combination of medications is prescribed, as is the case of ACS, should be assessed by considering all drugs together and using approaches that allow the identification of differential patterns of medication refill. However, no studies have examined adherence trajectories to multiple concurrent medications when combined therapy is recommended or have assessed their relationship with clinical outcomes. Thus, the objective of our study was 2-fold: first, to identify adherence patterns to combined recommended medication using GBTM, and second, to examine the association between adherence trajectories and subsequent cardiovascular events and mortality, in a cohort of patients with ACS.

## Materials and Methods

### Design

This study involved a population-based retrospective cohort of all patients discharged alive following an ACS from any Valencia Health System (VHS) hospital from January 2009 to December 2011. Patients were followed for 24 months from the date of hospital discharge (index date).

### Setting

The study was conducted in the region of Valencia in Spain, and specifically in the population covered by the VHS, with an extensive network of hospitals, primary care centers, and other facilities managed by the regional government, which provides universal free healthcare services (except for drug copayment) to 97% of the regional population (~5 million inhabitants, with 10% of the Spanish population).

### Population

We identified all patients aged 35 years and over who were discharged alive from VHS hospitals with the main diagnosis of ACS (ICD9-CM:410.xx—except 410.x2—and 411.xx) between January 2009 and December 2011, who had a prescription (filled or not) of three or more post-ACS prevention therapies (antiplatelet, beta-blockers, ACEI/ARB, and statins) within the first 30 days after discharge. Exclusion criteria were as follows: (1) deaths in the first year following hospital discharge (as this was the period to assess adherence); (2) people without pharmaceutical/health coverage by VHS; (3) non-residents, those who left the region, or who were discontinued from VHS coverage for other causes, due to limitations in follow-up.

### Data Sources

Data were obtained from the VHS Integrated Database (VID), which combines data sources linking them at an individual level through a single anonymized patient identifier. The main source of data was the ambulatory Electronic Medical Record, which includes information on diagnoses, personal medical history, laboratory test results, lifestyle factors, as well as information on both physician prescriptions and dispensations from pharmacy claims. The information on hospitalizations was based on the Minimum Basic Dataset (MBDS) at hospital discharge, and the synopsis of clinical and administrative information on all hospital discharges, including diagnoses and procedures. The Population Information System provides information on the population covered by the VHS and registers certain demographic characteristics, including the geographical location of each person and the dates and causes of VHS discharge, including deaths. A detailed description of the sources of data can be found elsewhere (24). The Ethics Committee of the Public Health General Directorate of the Valencia Health Authority and the Center for Public Health Research approved the protocol and waived the need for obtaining informed consent. All data used was pseudoanonymized.

### Clinical Outcome Measures

The three pre-specified clinical outcomes, measured within the second year of initial prescription of secondary prevention medications, were the first hospitalization for a major vascular event (acute myocardial infarction, unstable angina, stroke, or congestive heart failure) or coronary revascularization (identified by the following procedures: coronary bypass, stenting, or angioplasty); death by any cause (captured from the mortality registry); and a composite outcome of either a major vascular event, coronary revascularization or death. Only principal discharge diagnoses based on ICD9CM (Supplementary Table S1) were used to define endpoints. Out-of-hospital mortality was collected from the SIP system which, in turn, obtains the information from the mortality register. All outcomes were analyzed separately and only the first event was considered for analysis. Patients were followed from the start of the second year after the index event (day 366) until the end of that second year (day 730) or death, whichever came first.

### Drug Exposure: Adherence to Post-ACS Prevention Therapies

Based on electronic prescription and dispensation information, we constructed for each drug class a day of supply for the 365 days after the index date which indicates whether the medication was available or not on each day. The days of supply were estimated using the dosing regimen specified in the prescription (one tablet every 8, 12, or 24 h) and the number of pills per package/prescribed. If a dispensing occurs before the previous dispensing should have run out, days' supply was accumulated up to a maximum excess of 90 days of medication.

We first calculated a monthly PDC for the first 12 months after discharge for each patient's therapeutic group and created a binary indicator for “adherent” in each month defined as PDC ≥0.8 (i.e., 24 out of 30 days covered by medication). In the case of dual antiplatelet therapy, which is not recommended for all patients and with recommendations to be prescribed only for short-term (3, 5), we used a less restrictive approach, which required having at least one antiplatelet agent available to be classified as adherent to that group. To measure adherence to multiple concurrent drugs, we aggregated the monthly indicators of adherence to each therapeutic group and calculated a new binary indicator, identifying patients who were adherent to 3 or more medications each month.

Group-based trajectory models were used to identify and characterize different patterns of adherence to medication over time. This method is an application of finite mixture modeling that through maximum likelihood, identifies clusters of individuals and classifies individuals with similar trends and evolution of longitudinal measures (25, 26). We estimated logistic group-based trajectory models with 2 to 8 groups using a binary indicator of monthly adherence to three or more medications for the first 12 months as the longitudinal response, to model the probability of being adherent. We used until five-order polynomial in each model to allow the most flexibility to the trajectories. Model selection was based on the following parameters: (1) Bayesian information criterion (BIC) where the largest value indicates the best-fitting model, STATA traj module implementation: BIC = log(L) −0.5 ^*^ log(n) p, where L = log-likelihood, n = sample size and p = number of parameters); (2) a minimum proportion of the study sample in a class group or trajectory of 5%; (3) average posterior probability >0.7 in each group (25, 26). Additionally, we calculated the annual PDC for the first 12 months after discharge and defined adherence as having 80% of days covered by medication dispensed with 3 or more medications.

### Covariates

Covariates included relevant sociodemographic and clinical characteristics and measures of health service use at the time of discharge. We identified the following variables: main admission diagnosis (AMI, angina), age at hospital admission, gender, and copayment. Comorbidities included were the following: atrial fibrillation, congestive heart failure, chronic obstructive pulmonary disease (COPD), hyperlipidemia, hypertension, peripheral vascular disease, stroke, diabetes mellitus, dementia. Procedures at index hospitalization included the following: systemic or intracoronary thrombolysis, angiography, coronary artery bypass grafting (CABG), and percutaneous coronary intervention (PCI). Lifestyle included variables such as tobacco smoking and alcohol abuse. Additionally, we included the cardiovascular events that occurred during the year of adherence assessment for further adjustment. Health services use variables including outpatient visits, ED visits, and hospitalizations, as well as preventive medication use before the index date, and polypharmacy.

### Statistical Analysis

First, patients were classified according to their adherence patterns within the first year after discharge using GBTM. Then, the characteristics of patients were described according to each adherence trajectory group identified. Categorical variables were expressed as numbers and percentages, and continuous variables as mean and *SD*.

Incidence rates (per 100 person-year) for the clinical outcomes occurred within 365 days after the first-year treatment were calculated. Kaplan-Meier curves were plotted for each outcome. Multivariable Cox proportional hazards models were used to assess the association between trajectories of adherence to three or more secondary prevention medications and post-cardiovascular event and death within the 12 months after the first-year treatment exposure. We estimated a model for cardiovascular events and mortality independently, and another combining both events, whichever came first for the composite outcome. The model included all patient characteristics. Hazard ratios (HR) with their respective 95% CI were estimated for the final models.

Additionally, we performed a secondary analysis, in order to assess the robustness of our results, using the conventional adherence measure (e.g., annual PDC).

All analyses were performed using Stata 14 (StataCorp LP, College Station, TX, USA) and R 3.2.3 (R Foundation for Statistical Computing, Vienna, Austria) statistical software. The Traj module for Stata was used to run the GBTM (27).

## Results

### General Characteristics

**Figure 1:**
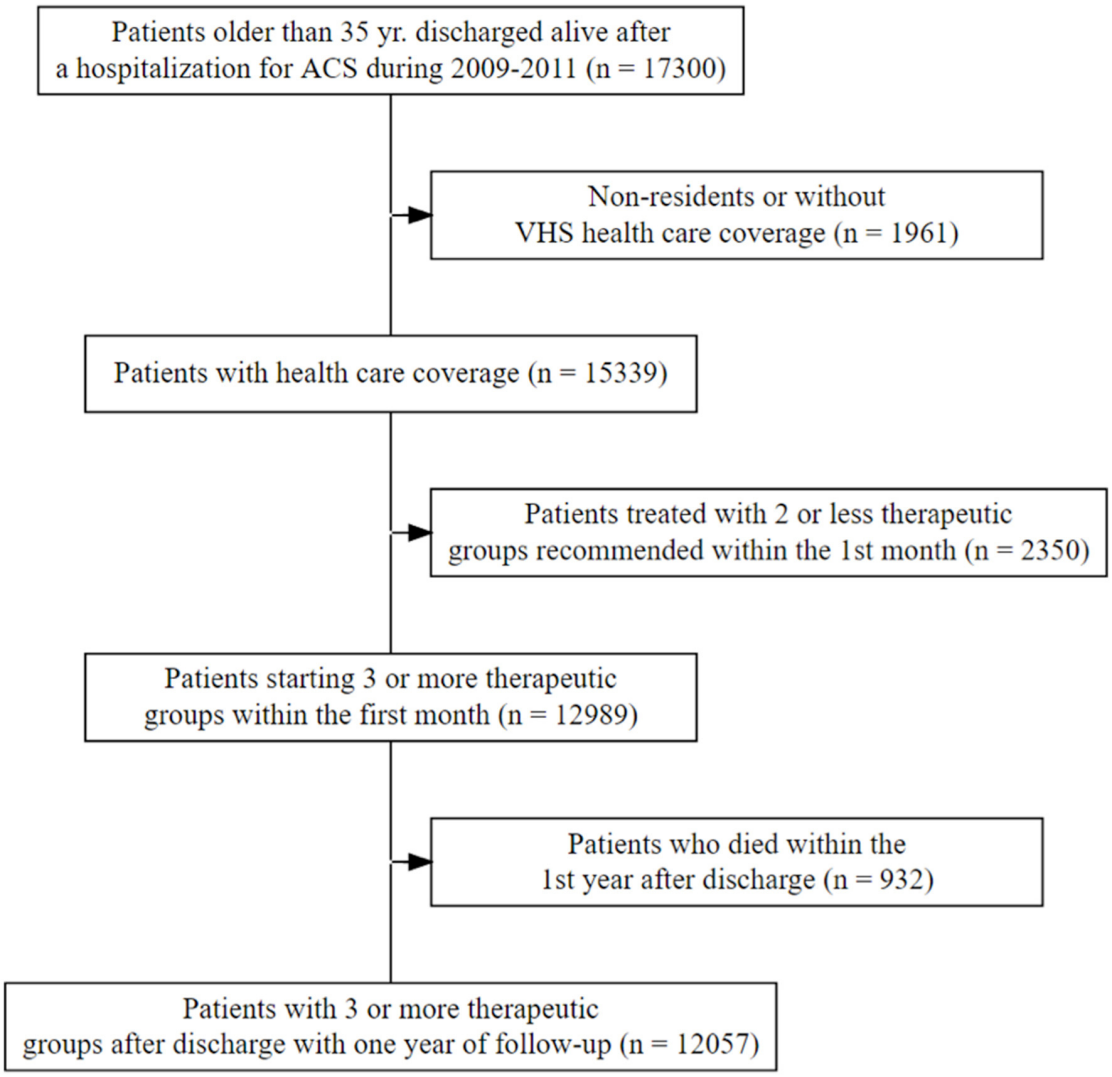
Flowchart of the population.

A total of 15,797 patients with ACS were discharged alive from 2009 to 2011. From these, 12,989 (82.2%) started treatment with ≥3 therapeutic groups during the first month after hospital discharge (Figure 1). Finally, a total of 12,057 (76.3%), who were prescribed at least 3 therapeutic groups during the adherence follow-up (i.e., 1 year after discharge), were kept for analysis. Of those, 98% were prescribed antiplatelets (78.1% had dual therapy), 82.1% beta-blockers, 82.4% ACEI/ARB, and 93.9% statins. Overall, 77.5% of patients had an acute myocardial infarction (AMI) as the main diagnosis of admission, 28% were women, and the median age was 68 years old. The average annual PDC for three or more medication therapies was 78.5 (*SD* 28.1; Median 92.1), and 66.4% of the cohort had a PDC ≥80% in the first year after hospital discharge (Table 1).

**Table 1:** Patient characteristics by adherence trajectory group.

**Characteristics**	**Adherent**	**Early gap**	**Middle gap**	**Late decline**	**Occasional users**	**Early decline**	**Non-adherent**	**Total**
*N*	6,383 (52.94%)	800 (6.64%)	684 (5.67%)	1,318 (10.93%)	657 (5.45%)	1,060 (8.79%)	1,155 (9.58%)	12,057 (100%)
**Age (%)**								
<45 years	3.95	3.38	3.95	5.16	5.02	9.34	5.45	4.72
45–64	35.47	36.62	40.94	40.44	43.99	40.66	37.84	37.55
65–79	44.37	42.88	41.08	37.56	35.46	31.60	35.06	40.84
80 and over	16.21	17.12	14.04	16.84	15.53	18.40	21.65	16.89
Female (%)	28.07	28.50	28.51	26.40	28.31	26.13	29.87	27.96
Copayment (%)	16.51	17.50	19.59	21.55	22.98	29.53	27.19	19.82
**Main diagnosis at discharge (%)**								
Acute Myocardial Infarction	80.54	71.50	77.05	75.64	73.36	77.08	70.39	77.54
Angina	19.46	28.50	22.95	24.36	26.64	22.92	29.61	22.46
**Medication use before hospitalization (%)**								
ACEI or ARB	53.64	59.50	53.51	50.38	50.84	41.60	46.84	51.80
Antiplatelet	33.98	49.38	40.06	39.53	41.55	30.75	38.44	36.51
Beta-blocker	23.58	36.00	23.25	28.60	31.05	19.81	23.55	25.01
Statin	43.16	53.75	47.51	46.97	46.88	35.09	40.78	43.79
**Comorbidity (%)**								
Hypertension	63.67	65.75	61.99	61.68	59.51	51.13	57.40	61.57
Diabetes	34.64	39.88	36.55	34.60	30.59	30.47	30.74	34.13
Lipid disorder	51.51	55.88	53.07	51.75	51.14	43.58	45.19	50.59
Congestive heart failure	6.74	11.62	7.60	7.66	11.42	8.40	10.39	7.96
Coronary heart disease	22.00	37.75	27.49	29.67	32.12	23.02	27.88	25.40
Arrhythmias	8.90	15.12	9.94	10.93	10.50	11.32	15.50	10.53
COPD	10.65	14.50	13.16	11.91	14.31	11.51	13.16	11.70
Chronic renal disease	4.07	6.50	4.53	5.39	6.09	5.38	7.97	5.00
Malignancy	8.15	8.62	6.87	6.37	8.52	5.38	7.10	7.59
Dementia	2.65	4.75	2.92	3.41	1.83	1.79	3.72	2.87
Stroke	10.97	13.00	14.33	13.81	10.05	9.53	13.51	11.67
Smoking	16.03	15.38	18.86	17.15	18.11	17.26	14.37	16.33
Alcohol	0.99	0.62	1.61	1.29	1.37	1.70	1.21	1.14
**Health care utilization, mean (SD)**								
No. ED visits	1.58 (1.35)	1.98 (1.89)	1.72 (1.40)	1.85 (1.71)	1.80 (1.65)	1.78 (1.61)	2.07 (2.22)	1.72 (1.58)
No. of Hospitalization visits	0.17 (0.55)	0.32 (0.74)	0.24 (0.69)	0.29 (0.75)	0.27 (0.69)	0.22 (0.67)	0.34 (0.87)	0.23 (0.66)
No. of outpatient physician visits	17.97 (16.90)	21.74 (21.55)	18.33 (17.69)	17.39 (16.72)	17.74 (18.80)	17.12 (19.85)	18.56 (20.44)	18.15 (18.03)
No. of prescription drugs	7.67 (5.37)	8.90 (5.72)	7.72 (5.61)	7.56 (5.52)	7.48 (5.67)	6.50 (5.79)	7.25 (5.85)	7.59 (5.55)
**Procedure on index hospitalization (%)**								
Angiography	49.60	44.12	49.42	45.68	47.64	46.04	46.93	48.12
Percutaneous coronary intervention	50.98	37.12	42.84	43.02	40.64	42.08	35.76	45.92
Coronary-artery bypass grafting	3.29	4.62	4.39	3.57	3.20	3.21	4.94	3.62
Systemic thrombolysis	10.03	6.50	8.92	7.21	8.83	9.15	6.67	8.96
Intracoronary thrombolysis	1.60	1.38	1.46	0.76	0.91	1.13	0.61	1.31
Length hospitalization, mean (SD)	8.82 (6.63)	9.15 (7.47)	8.92 (6.03)	8.59 (5.40)	8.32 (5.49)	8.87 (8.30)	9.88 (11.54)	8.90 (7.27)
Cardiovascular event during first year (%)	9.07	14.62	12.57	13.43	13.09	12.74	15.84	11.30
**Conventional measures of adherence**								
Annual PDC, mean (SD)	97.37 (3.54)	84.15 (7.77)	81.63 (9.58)	75.95 (11.85)	57.51 (14.75)	44.10 (15.00)	15.01 (14.38)	78.51 (28.08)
PDC >80 (%)	99.94	75.38	62.28	42.34	5.63	0.47	0.09	66.43

*ACEI, angiotensin-converting enzyme inhibitor; ARB, angiotensin receptor blocker; COPD, chronic obstructive pulmonary disease; ED, emergency department; SD, standard deviation; PDC, Proportion of days covered*.

### Adherence Trajectories

**Figure 2:**
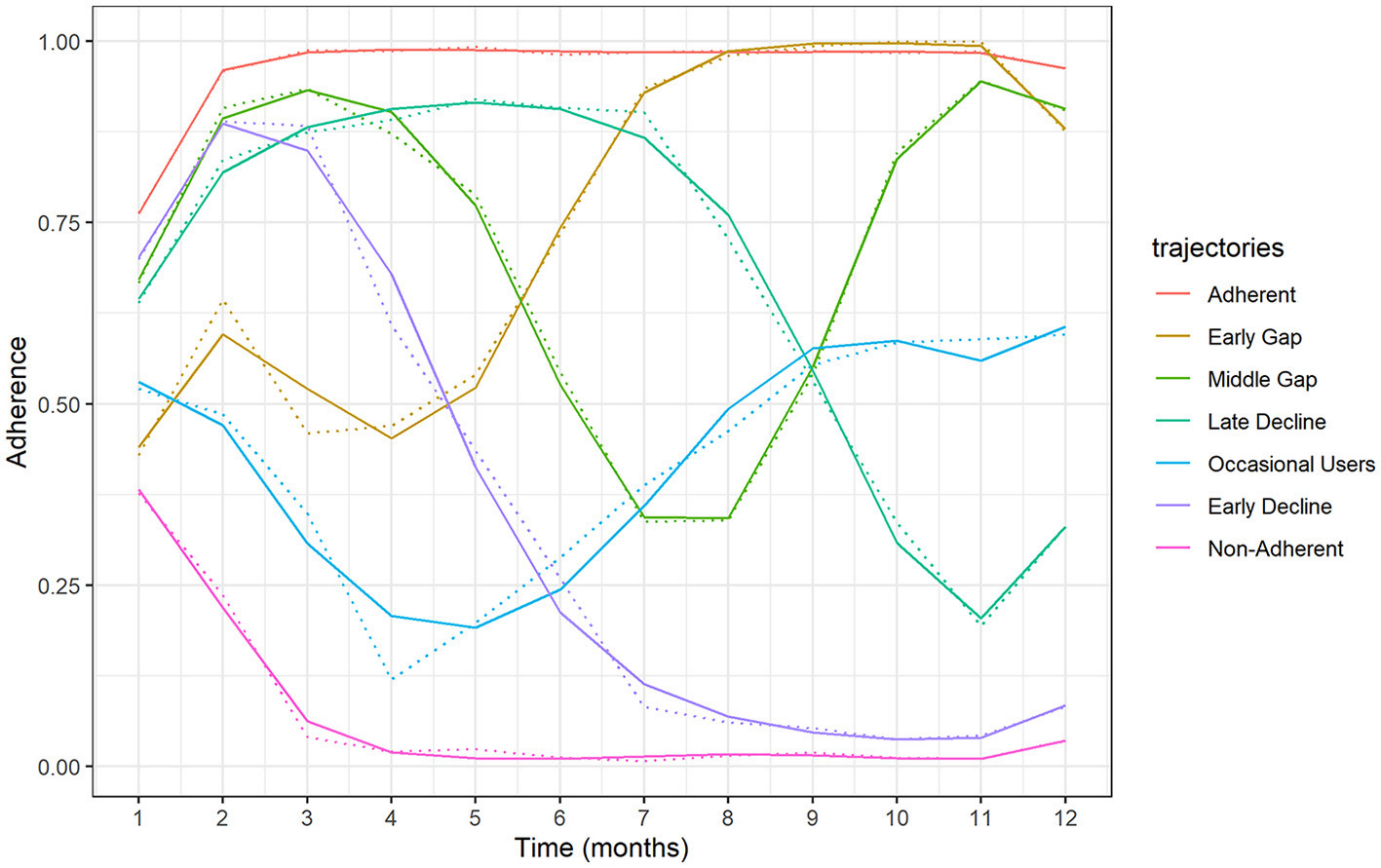
Adherence trajectories identified over the first year after an ACS.

In the GBTM analysis, we identified seven adherence trajectories after applying the pre-specified criteria. Adherence trajectories over the first year after an ACS are presented in Figure 2. These trajectories can be categorized into the following: (1) Adherent (52.94% of cohort patients); (2) Early Gap (6.64%); (3) Middle Gap (5.67%); (4) Late decline (10.93%); (5) Occasional Users (5.45%); (6) Early Decline (8.79%); (7) Non-Adherent (9.58%).

Adherent patients were less likely to have a copayment, cardiovascular comorbidities as well as chronic renal disease, ED and hospitalization visits, and were also less likely to have cardiovascular events during the first year after discharge but were more likely to have AMI as the main diagnosis at discharge and to have more revascularization procedures on the index hospitalization (Table 1).

Patients belonging to the early-gap group were less likely to be in the younger age category, and to report alcohol consumption, and showed higher use of medication before hospitalization, and were more likely to have most comorbidities studied (i.e., diabetes, lipid disorder, congestive heart failure, coronary heart disease, COPD, malignancy, and dementia).

The early decline group was more likely to have copayment and to be in the younger age category and showed lesser medication use before hospitalization in all therapeutic groups. It was also less likely to have comorbidities such as hypertension, diabetes, lipid disorder, chronic renal disease, malignancy dementia, and stroke.

Non-adherent patients were more likely to be aged 80 and over, to be female, and to have arrhythmias and chronic renal disease, they were also the second group most likely to have copayment (after the early-decline patients). Those belonging to this group also had more ED- and hospitalization visits, longer hospitalizations, and showed the highest percentage of cardiovascular events during the first-year post-discharge (Table 1).

### Clinical Outcomes

**Figure 3:**
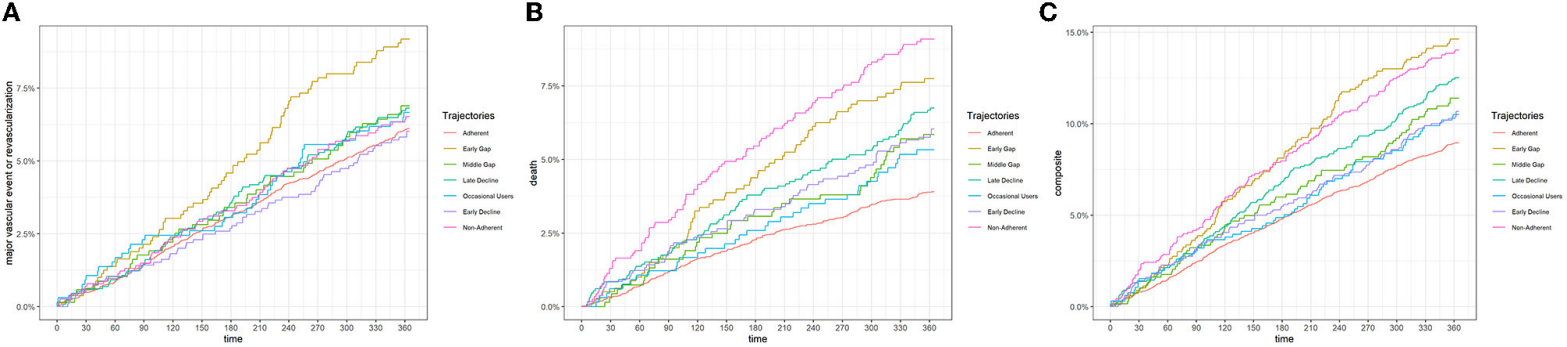
**(A)** Unadjusted incidence rates of major cardiovascular events. **(B)** Unadjusted incidence rates of death. **(C)** Unadjusted incidence rates of the composite outcome.

Regarding the unadjusted incidence rates of CV events, death, and the composite outcome for each adherence trajectory (Table 2, Figures 3A–C), no major differences were observed. The Early Gap trajectory showed the highest incidence of major vascular events or revascularization [9.65 per 100 person-year (p-y), 95% CI: 7.54, 12.17]. Regarding death, the non-adherent and the early-gap trajectories showed the highest rates (9.6 deaths per 100 p-y; 95% CI: 7.85, 11.62, and 8.1 deaths per 100 p-y; 95% CI: 6.21, 10.38, respectively). The adherent pattern showed the lowest incidence in the composite outcome (9.41 events per 100 p-y; 95% CI: 8.65, 19.06).

**Table 2:** Incidence rates (per 100 person-year) for adverse clinical outcomes.

	**Major vascular event or revascularization**			**Death**			**Composite**		
	**New cases**	**Person-years**	**Incidence rate (95%CI)**	**Deaths**	**Person-years**	**Incidence rate (95%CI)**	**New cases**	**Person-years**	**Incidence rate (95%CI)**
Adherent	383	6081.4	6.30 (5.68–6.96)	249	6245.8	3.99 (3.51–4.51)	572	6081.4	9.41 (8.65–10.21)
Early Gap	71	735.8	9.65 (7.54–12.17)	62	765.8	8.10 (6.21–10.38)	117	735.8	15.90 (13.15–19.06)
Middle Gap	46	644.0	7.14 (5.23–9.53)	40	664.1	6.02 (4.30–8.20)	78	644.0	12.11 (9.57–15.12)
Late Decline	87	1231.8	7.06 (5.66–8.71)	89	1271.2	7.00 (5.62–8.62)	165	1231.8	13.40 (11.43–15.60)
Occasional Users	43	621.5	6.92 (5.01–9.32)	35	639.6	5.47 (3.81–7.61)	69	621.5	11.10 (8.64–14.05)
Early Decline	62	1000.9	6.19 (4.75–7.94)	64	1026.2	6.24 (4.80–7.96)	113	1000.9	11.29 (9.30–13.57)
Non-Adherent	72	1063.7	6.77 (5.30–8.52)	105	1094.1	9.60 (7.85–11.62)	162	1063.7	15.23 (12.97–17.76)
Full cohort	764	11379.1	6.71 (6.25–7.21)	644	11706.8	5.50 (5.08–5.94)	1276	11379.1	11.21 (10.61–11.85)

*CI, confidence interval*.

**Figure 4:**
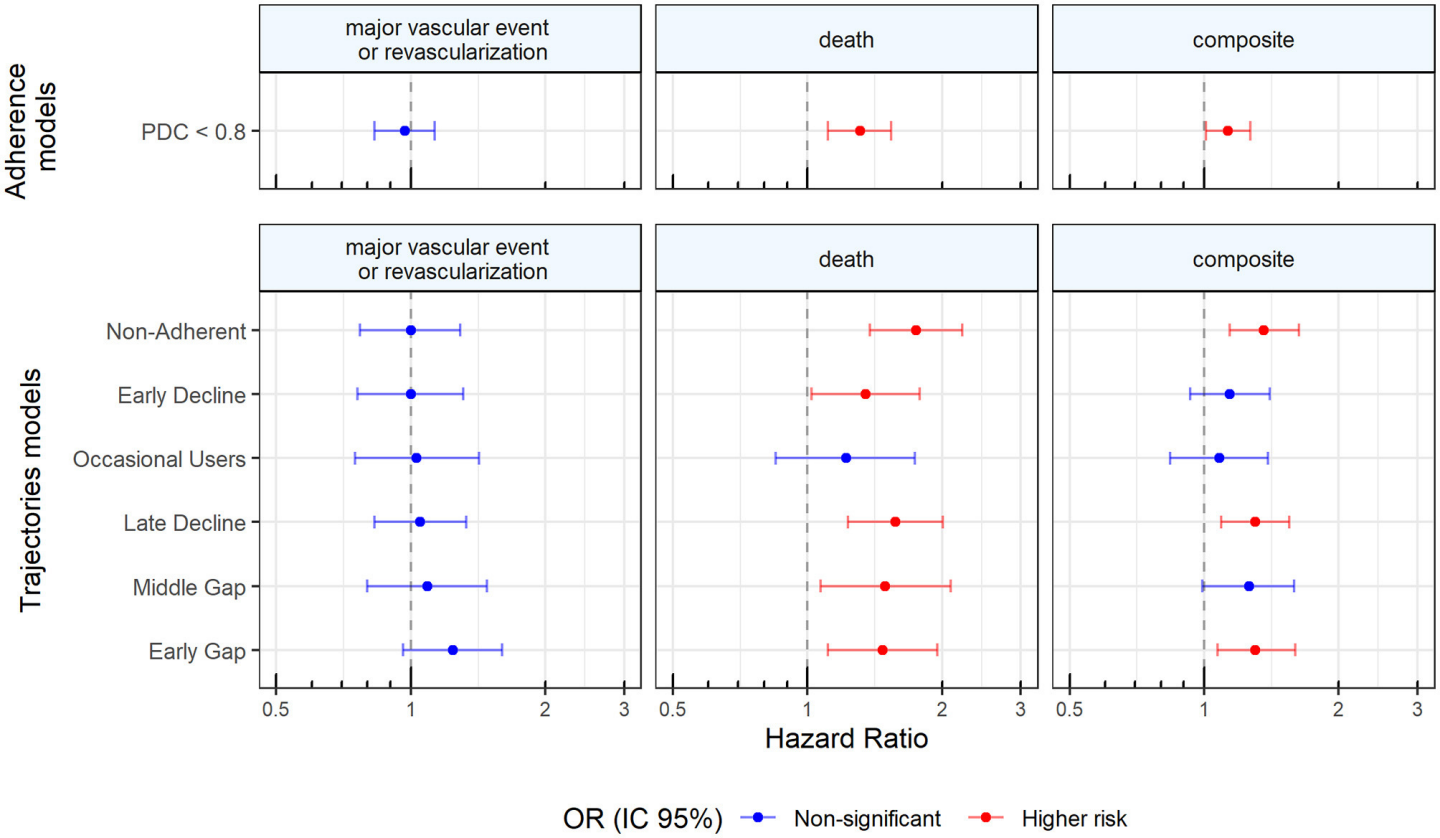
Adjusted association between adherence trajectories and clinical outcomes.

Figure 4 shows the adjusted association between adherence trajectories and clinical outcomes (see also Supplementary Table S2 for the full model). None of the trajectories was significantly associated with recurrence of major vascular events or revascularization in the adjusted analysis. Regarding mortality, compared to the adherent group, patients belonging to Middle-Gap (hazards ratio (HR): 1.5; 95% CI: 1.07, 2.09), Early-Gap (HR: 1.47; 95% CI: 1.11, 1.95), Late-decline (HR: 1.58; 95% CI: 1.24, 2.02), Early- Decline (HR: 1.35; 95% CI: 1.02, 1.79), and Non-Adherent (HR: 1.76; 95% CI: 1.38, 2.23) groups had a greater risk of mortality, being the non-adherent, the group with the highest likelihood of death. Similar associations were found for the composite outcome. Earl-Gap (HR: 1.3; 95% CI: 1.07, 1.6), Late-decline (HR: 1.31; 95% CI: 1.1, 1.56), and Non-Adherent patients (HR: 1.36; 95% CI: 1.14, 1.63) had a significantly greater risk of a major vascular event, revascularization or death as compared to Adherent patients.

When using dichotomized groups based on PDC, results were found to be robust, with the non-adherent patients being at higher risk of death or of presenting the composite outcome but showing no association with recurrent major vascular events (Figure 4, Supplementary Table S3). However, the risk found by using PDC < 80 was of lesser magnitude (HR: 1.31; 95% CI: 1.14, 1.54 for death and HR: 1.13; 95% CI: 1.01, 1.27 for the composite outcome) than those found in the poor adherence trajectories (Early Gap, Late Decline, and Non-Adherent; See Figure 4, Supplementary Tables S2, S3).

## Discussion

In the present study, we used group-based trajectory models to identify adherence patterns to three or more combined evidence-based medications in a concurrent manner in a population-based cohort of patients after hospitalization for acute coronary syndrome and evaluated which adherence patterns were associated with adverse clinical outcomes in the second year after discharge.

Although nowadays GBTM is not an uncommon tool to identify adherence patterns to medication over time using pharmacy claims databases (21–23, 28)_;_ to our knowledge, this is virtually the first time that adherence to multiple concurrent recommended medications is assessed through GBTM and compared to PDC estimates. GBTM accounts for the dynamic nature of adherence and allows the identification of differential behaviors in patients that otherwise would be classified simply as “non-adherent” (18–21). We identified seven adherence trajectories that best-summarized adherence patterns to three or more combined therapies for the secondary prevention of ACS. According to these trajectories, 47% of patients did not adhere to their treatment at some point in the first year post-ACS, but their patterns were very different, as were the baseline characteristics related to each pattern. *Non-adherence, Early gap*, and *Late Decline* trajectory groups were associated with a higher risk of the composite outcome of recurrent cardiovascular events, and all-cause death, compared to the adherent group. Furthermore, each of these patterns also showed differential risks (and of higher magnitude) when compared to PDC < 80 (the classical measure for non-adherent patients).

In a previous study of our group assessing adherence trajectories to the same medication groups, but individually, some of the patterns identified were similarly found here, including *adherent, early gap*, and *occasional users* (29). However, others differ from those shown in the present study. It could be possible that when accounting for the adherence to several drugs altogether, new patterns arise, as this represents a more complex scenario.

Through the patterns identified, we found that medication adherence was suboptimal, with 53% of participants belonging to the “Adherent” trajectory (sustained and continuous adherence) to ≥3 secondary prevention drugs. In the aforementioned study (29), we found that around 66% (for antiplatelet, beta-blockers, and ACEI/ARB) and 75% (for statins) of patients after hospitalization for coronary heart disease, were adherent (29). Again, differences could be explained; first, by the added complexity that means adhering to at least three different medications combined as a treatment, and second, by the different timespan in which adherence was assessed. These two factors can translate into findings of reduced adherence. The former factor is very important, as assessing drugs prescribed in combination, separately, could lead to overestimation of adherence. The two studies assessing 3 medications altogether: ACEI/ARB, beta-blockers, and statins (although using the traditional PDC or MPR) (15, 16), found that 49% and 34% of patients, respectively, adhere to all three therapies (15, 16). However, those figures are not directly comparable to our findings, as it does not provide information on adherence behaviors over time. For example, static PDC measures would classify patients into groups of adherent and non-adherent without differentiating the dynamics of the various non-adherent trajectories shown in our results. These differential behaviors are associated with different baseline characteristics and also differential risks for clinical outcomes as seen in our study. Identifying these features is key for targeting interventions aiming to improve adherence. For example, a modifiable factor, such as having copayment was found to be related to non-adherent trajectories. Other factors such as older age or female sex are indeed non-modifiable but can be considered when designing interventions.

We found that the adherence trajectories to ≥3 medications were associated with a higher risk of a composite outcome including major adverse cardiovascular events and death. We found an absence of a relationship between adherence trajectories and cardiovascular events alone, and such an absence of association was also observed in the analysis using PDC. These findings are likely to be due to the greater impact that adherence to preventive medication has in the acute phase post-ACS on the likelihood of a cardiovascular event. In fact, 11% of cohort patients experienced a cardiovascular event during the adherence assessment period and patients belonging to the Non-Adherent trajectory had the highest rate. On the other hand, our results show that increasing adherence to preventive therapies is associated with a significantly decreased risk of mortality in post-AMI patients similar to the previous study (10). It is important to note that outcomes were assessed during the second year after the index date, which is a common approach (15, 16). Therefore, we did not provide information regarding the impact of adherence to recommended therapies in the longer term, which should be further studied.

For the secondary prevention of acute coronary syndrome, clinical guidelines recommend all 4 therapies (antiplatelets, beta-blockers, ACE inhibitors/ARB, and statins) for long-term use for a major benefit. Hence, ideally, adherence to therapy when a combination of medications is prescribed should be assessed by considering all drugs together and using approaches that allow the identification of differential patterns of medication refill. This was the main focus of our study, and this principle could be applied to any other condition where combined therapy is recommended.

There were some limitations to this study. First, we used electronic prescription and dispensation information for measuring adherence that lacked information if dispensed medications were consumed by the patient. Second, we evaluated outcomes in the second year post-ACS and patients may have changed their adherence behavior to medication during this period. However, this is necessary to ensure the separation of our adherence and outcome measurement and to protect against bidirectional bias. Third, we only evaluated adherence for 12 months after hospitalization. Although non-adherence to secondary prevention post-ACS during the initial year of treatment is important, these medications are recommended for long-term use. Fourth, despite the availability of a wide range of potential confounders, unmeasured residual confounding by lack of adjustment for variables such as education or race and ethnicity cannot be ruled out.

## Conclusion

In this population-based study, we identified seven trajectories of adherence to three or more combined essential medications after ACS. Non-adherence, Early gap, and Late Decline trajectory groups were associated with a higher risk of the composite outcome of recurrent cardiovascular events and all-cause death, compared to the adherent group. Identification of patient subgroups with suboptimal adherence patterns may be useful to target interventions to improve medication adherence and health outcomes of patients when a therapy composed of several concurrent medications is recommended.

## Data Availability Statement

The datasets presented in this article are not readily available because we have no permission to make generated datasets available. We have been granted access to data by the Valencia Health Department, so we could make the analysis for this study, but are not allowed to share it. Requests to access the datasets should be directed to Unit of Analysis of Health Information Systems, Valencia Health Department (Servicio de Análisis De Sistemas de Información Sanitaria, Consellería de Sanitat). www.san.gva.es.

## Ethics Statement

The studies involving human participants were reviewed and approved by the Ethics Committee of the Public Health General Directorate of the Valencia Health Authority and the Center for Public Health Research approved the protocol and waived the need for obtaining inform consent. All data used was pseudoanonymized. Written informed consent for participation was not required for this study in accordance with the national legislation and the institutional requirements.

## Author Contributions

GS-G conceived the study. FS-S and DB-Q carried out the main statistical analyses. CR-B wrote the manuscript. IH prepared the database. CR-B and IH assisted with statistical analysis. CR-B, DB-Q, FS-S, IH, AG-S, SP, and GS-G participated in the study design and interpretation of data and contributed to the critical revision of the manuscript for important intellectual content. All authors agree to be accountable for all aspects of the work and have read and approved the final manuscript.

## Funding

This work was supported by the 2018 Health Outcomes Research Grant from Merck Health Foundation (partially) and by the Instituto de Salud Carlos III, Spanish Ministry of Health, through the REDISSEC network [grant number RD16/0001/0011 to CR-B, formerly and FS-S, currently]; and through a competitive grant [Sara Borrell CD19/00137 to CR-B, currently]. The funding sources have no access to study data, did not participate in any way in the design or conduct of the study, data analysis, or decisions regarding the dissemination of findings, the development of the manuscript, or its publication.

## Author Disclaimer

The views presented here are those of the authors and not necessarily those of the FISABIO Foundation, the Valencia Ministry of Health, or the study sponsors.

## Conflict of Interest

The authors declare that the research was conducted in the absence of any commercial or financial relationships that could be construed as a potential conflict of interest.

## Publisher's Note

All claims expressed in this article are solely those of the authors and do not necessarily represent those of their affiliated organizations, or those of the publisher, the editors and the reviewers. Any product that may be evaluated in this article, or claim that may be made by its manufacturer, is not guaranteed or endorsed by the publisher.
