# Supplementary material for: Assessing Concurrent Adherence to Combined Essential Medication and Clinical Outcomes in Patients With Acute Coronary Syndrome. A Population-Based, Real-World Study Using Group-Based Trajectory Models
Source: Front Cardiovasc Med. 2022 May 25;9:863876. doi: 10.3389/fcvm.2022.863876 (PMC9174582; doi:10.3389/fcvm.2022.863876)
Supplement: Supplementary file 1 [file Table_1.docx]

Supplementary Material

**Table S1.** International Classification of Disease, 9th edition, Clinical Modification (ICD-9-CM) codes used to define study clinical outcomes.

| **Diagnosis** | **ICD-9-CM codes** |
| --- | --- |
| Acute Myocardial Infarction | 410.xx excluding 410.x2 |
| Unstable ungina | 411.xx |
| Isquemic stroke | 433.01, 433.11, 433.21, 433.31, 433.81, 433.91, 434.01, 434.11, 434.91, 435, 435.x, 436, 437.1, 437.9 |
| Congestive Heart Failure | 428.xx |
| **Procedures** | **ICD-9-CM codes** |
| Coronary bypass | 36.1, 36.1x, 36.2 |
| Angioplasty | 00.66, 36.09 |
| Stent | 36.06, 36.07 |
| ICD-9-CM: International Classification of Diseases, 9th Revision, Clinical Modification. | |

**Table S2.**  Association of adverse clinical outcomes and adherence trajectory groups. Hazard ratios for the full cox model.

|  | **Major vascular event or revascularization** | **Death** | **Composite** |
| --- | --- | --- | --- |
| **Sociodemographic characteristics** | | | |
| age = p0 vs min HR age | 1.75 (0.84-3.66) | 1.29 (0.47- 3.57) | 1.56 (0.83- 2.91) |
| age = p25 vs min HR age | 1.03 (0.89-1.18) | 1.08 (0.84- 1.39) | 1.00 (1.00- 1.00) |
| age = p50 vs min HR age | 1.03 (0.92-1.15) | 1.65 (1.10- 2.48) | 1.14 (0.96- 1.36) |
| age = p75 vs min HR age | 1.19 (0.98-1.44) | 3.10 (2.08- 4.64) | 1.55 (1.28- 1.88) |
| age = p100 vs min HR age | 2.18 (0.94-5.06) | 20.13 (9.66-41.94) | 6.24 (3.60-10.83) |
| female | 1.04 (0.88-1.22) | 0.81 (0.67- 0.96) | 0.92 (0.81- 1.04) |
| copayment | 0.83 (0.62-1.11) | 0.98 (0.66- 1.47) | 0.88 (0.69- 1.12) |
| **Main Diagnosis at Discharge** | | | |
| acute myocardial infarction vs. angina | 1.14 (0.96-1.35) | 1.45 (1.20- 1.75) | 1.24 (1.08- 1.42) |
| **Medication use before hospitalization** | | | |
| Statin | 1.04 (0.86-1.26) | 0.97 (0.80- 1.19) | 1.02 (0.88- 1.18) |
| ACEI or ARB | 1.04 (0.86-1.25) | 1.06 (0.87- 1.28) | 1.05 (0.91- 1.21) |
| Beta-blocker | 1.28 (1.07-1.52) | 1.05 (0.87- 1.27) | 1.14 (1.00- 1.31) |
| Antiplatelet | 1.16 (0.96-1.40) | 1.16 (0.95- 1.41) | 1.24 (1.07- 1.43) |
| **Comorbidities** | | | |
| Hypertension | 0.97 (0.80-1.18) | 0.97 (0.78- 1.20) | 1.00 (0.85- 1.16) |
| Diabetes | 1.57 (1.34-1.83) | 1.51 (1.28- 1.79) | 1.47 (1.30- 1.66) |
| Lipid disorder | 0.95 (0.80-1.12) | 0.86 (0.72- 1.03) | 0.92 (0.81- 1.04) |
| Congestive heart failure | 1.28 (1.03-1.59) | 1.41 (1.14- 1.74) | 1.34 (1.14- 1.57) |
| Coronary heart disease | 1.17 (0.97-1.42) | 1.07 (0.88- 1.31) | 1.08 (0.93- 1.24) |
| Arrhythmias | 1.16 (0.95-1.42) | 0.97 (0.79- 1.19) | 1.08 (0.92- 1.25) |
| COPD | 0.98 (0.79-1.21) | 1.11 (0.90- 1.37) | 1.03 (0.88- 1.21) |
| Chronic renal disease | 1.48 (1.17-1.88) | 1.60 (1.28- 1.99) | 1.48 (1.25- 1.76) |
| Malignancy | 1.01 (0.78-1.29) | 1.28 (1.02- 1.60) | 1.18 (0.99- 1.40) |
| Dementia | 0.83 (0.56-1.24) | 1.61 (1.22- 2.12) | 1.32 (1.04- 1.66) |
| Stroke | 1.46 (1.22-1.76) | 1.19 (0.98- 1.44) | 1.30 (1.13- 1.49) |
| Smoking | 1.00 (0.81-1.23) | 0.77 (0.58- 1.01) | 0.94 (0.79- 1.12) |
| Alcohol | 1.19 (0.59-2.41) | 1.44 (0.53- 3.91) | 1.32 (0.74- 2.34) |
| **Health care utilization** | | | |
| No. ED visits | 1.02 (0.98-1.06) | 1.02 (0.98- 1.06) | 1.01 (0.98- 1.04) |
| No. of Hospitalization visits | 1.03 (0.93-1.14) | 1.15 (1.05- 1.25) | 1.09 (1.02- 1.17) |
| No. of outpatient physician visits | 1.00 (1.00-1.01) | 1.00 (1.00- 1.01) | 1.00 (1.00- 1.00) |
| No. of prescription drugs | 1.03 (1.01-1.05) | 1.02 (1.00- 1.04) | 1.03 (1.01- 1.04) |
| Length hospitalization | 1.00 (0.99-1.01) | 1.01 (1.00- 1.01) | 1.01 (1.00- 1.01) |
| Cardiovascular event during first year | 1.07 (0.87-1.30) | 1.82 (1.53- 2.18) | 1.45 (1.26- 1.66) |
| **Procedure on index hospitalization** | | | |
| Angiography | 1.01 (0.86-1.18) | 0.81 (0.67- 0.98) | 0.92 (0.81- 1.05) |
| Percutaneous coronary intervention | 0.97 (0.82-1.14) | 0.61 (0.50- 0.76) | 0.84 (0.74- 0.96) |
| Coronary-artery bypass grafting | 0.46 (0.26-0.81) | 0.42 (0.22- 0.81) | 0.46 (0.30- 0.70) |
| Systemic or intracoronary thrombolysis | 0.87 (0.65-1.17) | 0.72 (0.49- 1.06) | 0.87 (0.69- 1.10) |
| **Adherence Trajectories (vs Adherent trajectory)** | | | |
| Early Gap | 1.24 (0.96-1.60) | 1.47 (1.11- 1.95) | 1.30 (1.07- 1.60) |
| Middle Gap | 1.09 (0.80-1.48) | 1.49 (1.07- 2.09) | 1.26 (0.99- 1.59) |
| Late Decline | 1.05 (0.83-1.33) | 1.57 (1.23- 2.01) | 1.30 (1.09- 1.55) |
| Occasional Users | 1.03 (0.75-1.42) | 1.22 (0.85- 1.74) | 1.08 (0.84- 1.39) |
| Early Decline | 1.00 (0.76-1.31) | 1.35 (1.02- 1.78) | 1.14 (0.93- 1.40) |
| Non-Adherent | 1.00 (0.77-1.29) | 1.75 (1.38- 2.22) | 1.36 (1.14- 1.63) |
| **Discrimination measure** | | | |
| c-index | 0.697 | 0.819 | 0.737 |
| optimism-corrected c-index | 0.680 | 0.810 | 0.729 |
|  |  |  |  |

COPD = Chronic obstructive pulmonary disease; PDC = Proportion of days covered. Age is introduced in the model as pspline of 4 df. HR of age are represented as the HR between p0, p25, p50, p75 and p100 ages vs the age that minimizes the HR. Age percentiles p0, p25, p50, p75 and p100 are 35, 57, 68, 77 and 99 years respectively. Age that minimizes HR for each model from left to right are 63, 50 and 57 years respectively

**Table S3.** Association of adverse clinical outcomes and conventional adherence. Hazard ratios for the full cox model.

|  | **Major vascular event or revascularization** | **Death** | **Composite** |
| --- | --- | --- | --- |
| **Sociodemographic characteristics** | | | |
| age = p0 vs min HR age | 1.74 (0.83-3.64) | 1.28 (0.46- 3.54) | 1.55 (0.83- 2.90) |
| age = p25 vs min HR age | 1.03 (0.90-1.18) | 1.08 (0.84- 1.39) | 1.00 (1.00- 1.00) |
| age = p50 vs min HR age | 1.03 (0.92-1.15) | 1.65 (1.10- 2.48) | 1.14 (0.96- 1.35) |
| age = p75 vs min HR age | 1.18 (0.97-1.43) | 3.11 (2.08- 4.65) | 1.55 (1.28- 1.87) |
| age = p100 vs min HR age | 2.16 (0.93-5.01) | 19.89 (9.63-41.11) | 6.20 (3.59-10.72) |
| female | 1.04 (0.88-1.22) | 0.81 (0.68- 0.97) | 0.92 (0.81- 1.04) |
| copayment | 0.83 (0.62-1.11) | 1.02 (0.68- 1.52) | 0.89 (0.70- 1.14) |
| **Main Diagnosis at Discharge** | | | |
| acute myocardial infarction vs. angina | 1.14 (0.96-1.35) | 1.43 (1.18- 1.72) | 1.23 (1.07- 1.40) |
| **Medication use before hospitalization** | | | |
| Statin | 1.05 (0.87-1.26) | 0.97 (0.79- 1.19) | 1.02 (0.88- 1.18) |
| ACEI or ARB | 1.04 (0.86-1.24) | 1.05 (0.86- 1.28) | 1.04 (0.91- 1.20) |
| Beta-blocker | 1.28 (1.07-1.53) | 1.05 (0.87- 1.27) | 1.14 (1.00- 1.31) |
| Antiplatelet | 1.16 (0.96-1.41) | 1.17 (0.96- 1.43) | 1.25 (1.08- 1.44) |
| **Comorbidities** | | | |
| Hypertension | 0.97 (0.80-1.18) | 0.96 (0.77- 1.19) | 0.99 (0.85- 1.16) |
| Diabetes | 1.57 (1.34-1.83) | 1.50 (1.27- 1.78) | 1.47 (1.30- 1.66) |
| Lipid disorder | 0.94 (0.80-1.11) | 0.87 (0.72- 1.04) | 0.92 (0.81- 1.04) |
| Congestive heart failure | 1.28 (1.03-1.59) | 1.38 (1.12- 1.71) | 1.32 (1.13- 1.55) |
| Coronary heart disease | 1.18 (0.98-1.42) | 1.08 (0.88- 1.31) | 1.08 (0.94- 1.25) |
| Arrhythmias | 1.17 (0.96-1.43) | 0.98 (0.80- 1.21) | 1.09 (0.94- 1.27) |
| COPD | 0.98 (0.79-1.21) | 1.13 (0.91- 1.39) | 1.04 (0.89- 1.22) |
| Chronic renal disease | 1.48 (1.17-1.87) | 1.60 (1.28- 1.99) | 1.48 (1.24- 1.76) |
| Malignancy | 1.01 (0.79-1.29) | 1.28 (1.02- 1.61) | 1.18 (0.99- 1.40) |
| Dementia | 0.84 (0.56-1.24) | 1.61 (1.22- 2.12) | 1.33 (1.05- 1.68) |
| Stroke | 1.46 (1.22-1.76) | 1.20 (0.99- 1.46) | 1.31 (1.13- 1.50) |
| Smoking | 1.00 (0.81-1.23) | 0.76 (0.58- 1.01) | 0.94 (0.79- 1.12) |
| Alcohol | 1.19 (0.59-2.41) | 1.45 (0.53- 3.92) | 1.32 (0.74- 2.36) |
| **Health care utilization** | | | |
| No. ED visits | 1.02 (0.98-1.07) | 1.02 (0.98- 1.07) | 1.01 (0.98- 1.05) |
| No. of Hospitalization visits | 1.03 (0.93-1.14) | 1.15 (1.06- 1.25) | 1.09 (1.02- 1.17) |
| No. of outpatient physician visits | 1.00 (1.00-1.01) | 1.00 (1.00- 1.01) | 1.00 (1.00- 1.00) |
| No. of prescription drugs | 1.03 (1.01-1.05) | 1.01 (1.00- 1.03) | 1.03 (1.01- 1.04) |
| Length hospitalization | 1.00 (0.99-1.01) | 1.01 (1.00- 1.02) | 1.01 (1.00- 1.01) |
| Cardiovascular event during first year | 1.07 (0.88-1.31) | 1.83 (1.53- 2.19) | 1.46 (1.27- 1.68) |
| **Procedure on index hospitalization** | | | |
| Angiography | 1.01 (0.86-1.18) | 0.81 (0.67- 0.98) | 0.92 (0.81- 1.05) |
| Percutaneous coronary intervention | 0.96 (0.82-1.13) | 0.61 (0.49- 0.75) | 0.83 (0.73- 0.95) |
| Coronary-artery bypass grafting | 0.46 (0.26-0.81) | 0.42 (0.22- 0.81) | 0.46 (0.30- 0.70) |
| Systemic or intracoronary thrombolysis | 0.87 (0.65-1.16) | 0.71 (0.49- 1.04) | 0.86 (0.68- 1.09) |
| **Clasical Measures of Adherence** | | | |
| PDC < 0.8 | 0.97 (0.83-1.13) | 1.31 (1.11- 1.54) | 1.13 (1.01- 1.27) |
| **Discrimination measure** | | | |
| c-index | 0.696 | 0.818 | 0.736 |
| optimism-corrected c-index | 0.682 | 0.809 | 0.729 |

*Note:* COPD = Chronic obstructive pulmonary disease; PDC = Proportion of days covered. Age is introduced in the model as pspline of 4 df. HR of age are represented as the HR between p0, p25, p50, p75 and p100 ages vs the age that minimizes the HR. Age percentiles p0, p25, p50, p75 and p100 are 35, 57, 68, 77 and 99 years respectively. Age that minimizes HR for each model from left to right are 63, 50, and 57 years respectively.
